# The Type of Diseases in Middle Georgia

**Published:** 1876

**Authors:** J. C. C. Blackburn

**Affiliations:** Barnesville, Ga.


### THE TYPE OF DIEASES IN MIDDLE GEORGIA.

BY J. C. C. BLACKBURN, M. D., BARNESVILLE, GA.

*Read before the M. G. M. Society, [illegible]ov. 17 [illegible]875 and requested to be published.*

It has been my purpose for a length of time to give an account of the particular type of diseases which have occurred in this section of the State, during the years 1846 to 1851, 1859 to 1865, and 1872 to 1875, embracing the periods of my practice at this point. Having kept a case book all along through these periods, I shall only give facts as culled from that source, without entering into a detailed account of concomitant circumstances, which may, or may not have modified the peculiarities, which I shall briefly endeavor to state. In 1846 I located at Barnesville, Pike county, Ga., for the purpose of engaging in the practice of medicine. Dr. W. H. Holley, a physician famous for his personal peculiarities and good common sense, being my only competitor. My practice extended over parts of Pike, Monroe, Upson and Butts counties, which area was in the middle beat of the State. The principal streams of water permeating this district were Potato creek, Tobler creek, and Towaliga rivers, (little and big). The country was then sparsely settled, and dams across the streams which obstructed their course, few and far between. The population were sturdy, athletic laborers, and consequently few "fashionable" diseases were known- When I say fashionable, I mean those diseases which are the result of refined life, such as indigestion, neuralgia, (the result of irregularities), and kindred affections. I shall confine my remarks to the old type of fevers, described by Eberle, Thacker, Rush, Chapman, and other authors of that day, under the terms Synochus and Synochal. The spring of 1846 ushered in pleurisy very prominently indicated by the " pleurisy stitch " in the right side, which almost invariably yielded to the lancet, an emetic, followed by the old "ten and ten"—jalap and calomel. In the summer we had bilious fever of synochal, dynamic, or inflammatory type. This from the derivation of the words " sunicho, "

"sun," and "echo," I hold, I continue, will naturally indicate that the fever was *continued.* A brisk cathartic, preceded by free venesection, followed by quinine, or *the bark*, generally stereotyped the treatment, and generally terminated, when taken at its incipiency, successfully. In the fall of that year we had scarcely any sickness, except near mill-ponds or water courses, which was of an intermittent type. Strange to say that although the type of fever had been in the summer strictly synochal, in the fall of this year, and although either intermitting or strictly remitting, it generally was so intermingled that it was often hard to determine its' distinguishing features. Here was the first advent of a peculiar type of fever, which was afterwards to assume the fearful concomitants of typhus or typhoid fevers. As I remarked in regard to the fever of the preceding summer, unless this fall fever was attacked vigorously at the start, it assumed a low grade, and despite the three *herculean doses of five grains of quinine* at a dose, became protracted and difficult to control. The eonvalescence from these fevers was tedious, and relapses common. In 1847, we had scarcely any of the preceding types of disease. Scarlet fever, very malignant, and dysentery prevailed extensively during the spring and summer months. At the advent of the fever I instituted a stimulant treatment, avoiding any thing that would deplete the system, and my success verified the correctness of the course adopted. In treating dysentery, I found it to be of a bilious type, and relied, in the main, in its management, upon calomel, ipicac and opium. In the fall of this year, which, in my district, was peculiarly exempt from disease, we had an occasional attack of remittent, and from my case book, no intermittent fever. It was mild and easily controlled. In 1848 we had pneumonia in the spring, followed by diarrhea, which culminated in the summer in *continued* fever. Here, so far as my observation extends, was truly the natal day of typhoid fever, or its counterpart, a low grade of remitting fever, which so closely assimilated the former that it was oftentimes hard to distinguish between them.

It did not then, as often now, seem to have been controlled by malarious influences, but was *sui generis*, a form peculiar to itself. Remembering that diarrhea had just preceded it, I concluded that cathartics might prove dangerous, hence, in its treatment I administered this class of remedies very cautiously. My treatment was at its commencement to relieve the *prima via* of suspected irritating matter, give an anodyne, generally Dovers powder, at night to fortify the patient for the events of the succeeding day, and combat symptoms as they might arise thereafter. I was unfortunate in the management of cases which occurred in my practice, losing at least one-half of the cases of well developed type. In 1849, we had nearly the same class of fevers as occurred in the two years previous, with the exception of dysentery, which, from its inception, was inflammatory, and seemed not to be complicated, with derangement of the liver, or congestion of the portal system. The use of the lancet freely at the start, cupping and application of leeches, acccompanied with anodynes, discarding strictly astringent remedies, marked my treatment of this disease, and I believe the result verified the conclusion arrived at. From 1860 to 1865, embracing the war, most of which time I had no competitors or aid in this locality, diseases were rare and their treatment easy. Indeed, Providence, as I then thought, seemed to smile upon our people, and in the absence of remedial agents, which were hard to procure, our people seemed to get well with little or no physic. I reflected upon this fact seriously, and at length reached the conclusion that ordinarily too much physic had been given, and resolved to shape my future practice accordingly.

Term 1872 to 1875—We have had a marked tendency to congestion, either of stomach or bowels, in any case of fever that I have seen. This year has presented this distinguishing peculiarity more plainly than I ever before noticed. My confreres will endorse the assertion that, in scarcely any case of fever treated, were we ever called upon to combat either developed or threatened congestion of those organs. I can safely say that in one hundred cases of fever visited and treated by me this year, congestion of stomach and bowels, either latent or extant, was met with. As regards the treatment of fever this year, quinine, unless given in large abortive doses, (say 20 to 50 grs. at a dose), at the very commencement of the fever, has been productive of but little good. Indeed, I am inclined to the opinion from over a quarter of a century's experience, and a close observation of the therapeutical effects of this drug, that in the fevers of Middle Georgia to prove potent, it should be given in large doses. I formerly treated "chills and fever" by giving from five to ten grains during intermission. Now I never give less than ten grains at once during intermission, and repeat in six hours, and never fail to break down an ordinary case of chills and fever. In congestive types of fever, a physician might as well attempt to tickle a storm with a feather, as to administer quinine in five grain doses with a view of relieving or preventing congestion. I have given this season, over *one hundred* grains of quinine in twelve hours, and been rewarded by the recovery of the patient, and in no case where I have administered large doses have I been disappointed in its expected effect. I will add here, by way of parenthesis, that I have never yet seen a solitary case of deafness result from the use of this chemical in my hands.

These hasty reflections from a series of years of a laborious country practice, are given, in rough, unvarnished style, as such observations must needs be, gathered, as they have been, from a case book, detailing the history and treatment of disease as it occurred in my practice, written impulsively, without the least idea of ever referring to it for an article for the *Medical Journal*, hoping that what has been written will escape severe criticism on the one hand, and stimulate every young physician to the keeping of a regular diary of his professional labors. Thirty years practice in the profession of my choice, to which I have devoted the best energies of mind and strength, warn me that I have but poorly discharged its responsible duties. I look back upon the past, and see many brilliant opportunities neglected, many bright prospects blackened, and, in the survey of life, can but exclaim, that after many years of study and active investigation, like one of old, "I seem to be standing on the shores of the great ocean of truth, gathering here and there a pebble."

